# Development of a patient journey map for people living with cervical dystonia

**DOI:** 10.1186/s13023-022-02270-4

**Published:** 2022-03-21

**Authors:** Monika Benson, Alberto Albanese, Kailash P. Bhatia, Pascale Cavillon, Lorraine Cuffe, Kathrin König, Carola Reinhard, Holm Graessner

**Affiliations:** 1 Dystonia Europe, Brussels, Belgium; 2 European Reference Network for Rare Neurological Diseases, Tübingen, Germany; 3 Department of Neurology, IRCCS Istituto Clinico Humanitas, Rozzano, MI 20089 Italy; 4 Department of Clinical and Movement Neuroscience, Institute of Neurology UCL, London, WC1N 3BG UK; 5 Patient Centricity, Ipsen, Boulogne Billancourt, France; 6 Medical Affairs, Ipsen, Slough UK; 7 PARTNERSEITZ GmbH, Ludwigshafen, Germany; 8 Centre for Rare Diseases and Institute of Medical Genetics and Applied Genomics, University Hospital Tübingen, Calwerstr. 7, 72076 Tübingen, Germany

**Keywords:** Cervical dystonia, Patient journey, Rare disease, Patient survey, Patient communication, Access to treatment

## Abstract

**Background:**

Patient journey maps are increasingly used as a tool that enables healthcare providers to refine their service provision to best meet patient needs. We developed a cervical dystonia patient journey map (CDPJM) that describes the holistic patient experience from pre-diagnosis through to long-term treatment.

**Methods:**

The CDPJM was developed in 2 stages; a patient survey (open questions and multichoice) of 15 patients with CD was conducted to inform the design of the CDPJM, which was then refined and validated by an expert-patient focus group.

**Results:**

Qualitative analysis of the patient survey supported five key stages of the patient journey: symptom onset, diagnosis and therapeutic relationship with healthcare professionals, initiation of care for CD, start of CD treatment, and living with treated CD. Following symptom onset, survey respondents described having multiple visits to their family doctor who prescribed strong pain killers and muscle relaxants and referred their patient to up to 10 different specialists for diagnosis. Over half (53.3%) of respondents had received ≥ 1 misdiagnosis. Respondents reported relief at having a diagnosis but a lack of understanding of the prognosis and treatment options; 46.7% said their neurologist did not spend enough time addressing their concerns. Survey respondents reported using a variety of alternative sources of information, including the internet (86.7%), self-help groups (66.7%) and information leaflets provided by health care professionals (60.0%). While botulinum toxin (BoNT) was consistently discussed as the main treatment option, some neurologists also mentioned physiotherapy, counselling, and other complementary approaches. However, patients were often left to seek complementary services themselves. Patients reported a ‘rollercoaster’ of relief with BoNT treatment with symptoms (and subsequent impact on daily life) returning towards the end of an injection cycle. *“When BoNT works well I can return to an almost normal life … when the injections stop working so well, I have to rest more and avoid going to work and experience life restrictions.”*

**Conclusions:**

We present the first patient journey map for CD that can be used to guide local service mapping and to compare current provision with what patients say they want and need.

**Supplementary Information:**

The online version contains supplementary material available at 10.1186/s13023-022-02270-4.

## Background

Cervical dystonia (CD) is a is focal dystonia of the cervical region primarily characterized by involuntary contractions of the neck muscles, resulting in twisting and repetitive movements, and abnormal postures of the head. CD may also present with tremor [1, 2]. It is one of the most common forms of adult-onset dystonia with a recent estimated incidence of about 1.18 per 100,000 person-years [3]. The average age of CD onset is around 41 years old [4, 5], and many patients are working and have young families when they are diagnosed [6]. Disability with functional impairment, pain and embarrassment with social withdrawal are common and bring significant quality of life burdens [7–9]. Treatment with botulinum toxin (BoNT) injections is considered first line therapy [10, 11].

In recent years there has been a shift towards ‘patient engagement’, broadly defined by World Health Organization (WHO) as *“the process of building the capacity of patients, families, carers, as well as health care providers, to facilitate and support the active involvement of patients in their own care, in order to enhance safety, quality and people-centeredness of health care service deliver”’* [12]. However, such a shift requires an understanding of the patient experience [13], and while there have recently been some important patient surveys to better understand how CD and its management impacts patients, they have tended to focus on daily burden [14] and specific aspects of CD management [15, 16]. Another way to visualize the patient experience is to develop a patient journey map, which describes the processes that patients go through when they undergo diagnosis and treatment. This consists of several stages, where each stage comprises one or more healthcare touchpoints [17]. The insights gained from the patient mapping process can help a service designer optimize the experience and generate value for both the user and the healthcare organization providing the service.

Patient journey maps are increasingly used as a tool that enables healthcare providers to reconfigure their approach to the treatment and care, seen from the patients’ point of view [17–19]. Through patient journey mapping, a healthcare provider and other stakeholders can identify unmet needs, the barriers and potential gaps in service provision, and work on the solutions to these problems, as well as identifying potential new opportunities for improvement and innovation [17, 19]. Additionally, patient journey tools are increasingly used as a baseline for designing and improving treatment algorithms and developing costing models that can be used to audit the impact of service improvements [20]. As part of the ongoing European Reference Networks for Rare Neurological Diseases (ERN-RND) program [21–23], we aimed to develop a patient journey map for CD that describes the patient experience from pre-diagnosis through to long-term treatment. The CD patient journey map (CDPJM) is presented from the perspective of a 'typical’ patient (Lilly), a persona developed based on the feedback of the patient survey.

## Results

The CDPJM was developed between March and June 2021 by a patient experience research company (PARTNERSEITZ) in collaboration with patient representatives from Dystonia Europe and affiliated national societies, and was sponsored by Ipsen. The CDPJM was developed in two stages. First, a patient survey of 15 patients living with CD was conducted to inform the design of the map, and secondly, an expert-patient focus group met to review and validate the map and suggest any refinements.

### Online patient survey

Fifteen patients living with CD (five each from France, Italy, and the UK) completed the online patient survey between the 24^th^ and 31^st^ March 2021. Key respondent characteristics are presented in Table 1; three quarters of respondents were female and the mean age at diagnosis was 41.5 years. All of the survey respondents were living with chronic CD (≥ 5 years), with 53.3% having been diagnosed more than 10 years ago.

**Table 1:** Respondent characteristics for patients completing the online survey

Characteristic	Survey response
Female/male, n (%)	12/3 (80%/20%)
Age (years), mean ± SD	54.1 ± 10.9
Age at diagnosis (years), mean ± SD	41.5 ± 9.6
Employed, n (%)	8 (53.3%)
Time since diagnosis, n (%)	
Within past 5 years	2 (13.3%)
Within past 5–10 years	5 (33.3%)
> 10 years ago	8 (53.3%)
First symptoms experienced at onset*	
Head and/or neck tilting/twisting	11 (73.3%)
Neck spasms	3 (20.0%)
Pain	6 (40.0%)
Tremor	5 (33.3%)

^*^Patients cited the first symptoms they recalled experiencing at onset (open question allowing multiple symptoms to be identified)

**Fig. 1:**
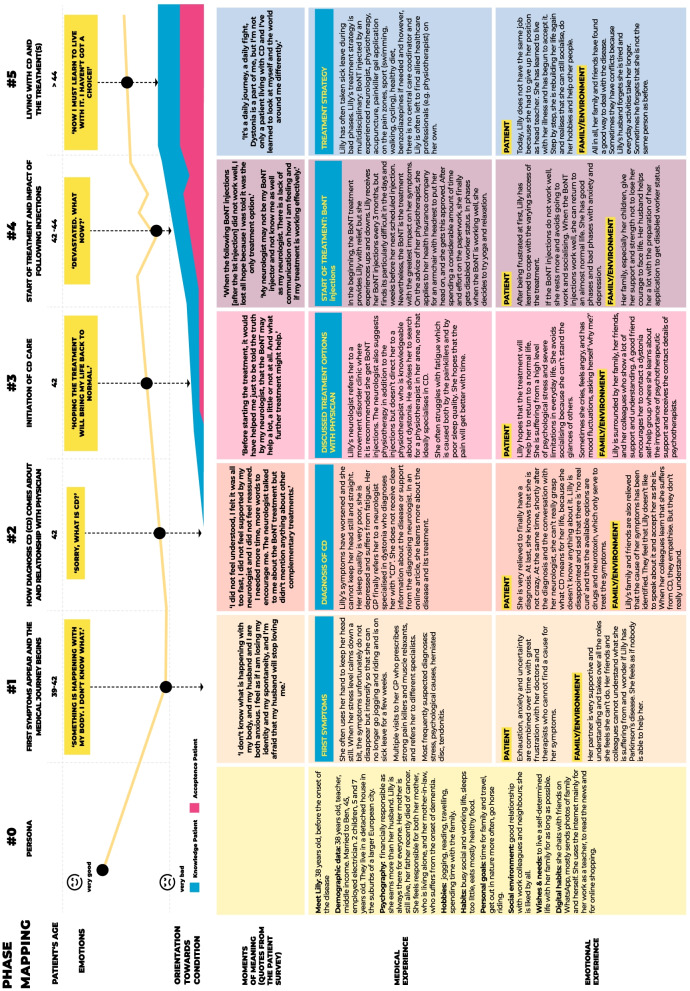
Abbreviated CD patient journey map

Figure 1 shows an abbreviated version of the CDPJM. The full version is given in Additional file 1. Qualitative analysis of the patient survey supported five key stages of the patient journey:

1. Symptom onset
2. Diagnosis and therapeutic relationship with healthcare professional (HCPs)
3. Initiation of care for CD
4. Start of CD treatment
5. Living with treated CD

At symptom onset, most survey respondents (n = 12, 80%) reported abnormal head and/or neck positions as their first symptom of CD. The other three respondents reported tremor (n = 2) and pain (n = 3) as their earliest symptoms. At this time, 60% of survey respondents already described impact on their daily activities (e.g., eating, drinking, walking, any physical activities) and 10 (66.7%) reported an impact on sleep. Survey respondents described having multiple visits to their family doctor who frequently prescribed strong pain killers and muscle relaxants, and referred their patient to various specialists including neurologists, rheumatologists, orthopedists, psychologists, radiologists, physiotherapists and chiropractors. Misdiagnosis was common (53.3% of survey respondents had received ≥ 1 misdiagnosis) with suspected diagnoses including: stress and other psychological causes, muscular sclerosis, herniated discs, tendonitis and stiff neck due to air conditioning. Without a diagnosis, survey respondents cited feeling even more anxious and stressed, and shame/embarrassment about their condition which was often obvious to their family, friends, and co-workers.

**Fig. 2:**
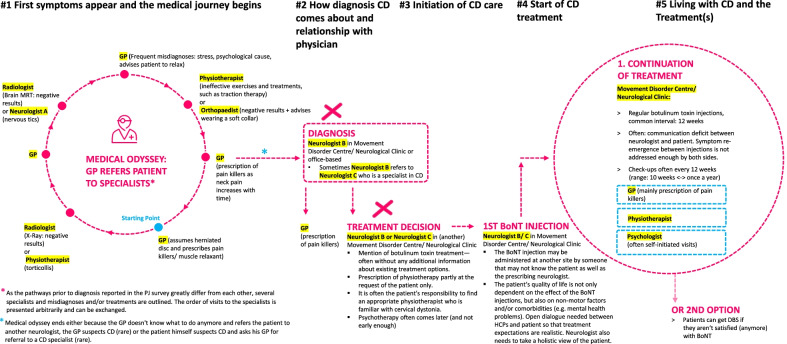
The intersectoral pathway

By the time of diagnosis and initiation of CD care, survey respondents had already seen up to 10 specialist and non-specialist HCPs [range 1–10] before being diagnosed with CD by a neurologist. Figure 2 shows a typical intersectoral pathway, from diagnosis through to treatment. Some of the respondents described meeting more than one neurologist before receiving their diagnosis, with many respondents finally being referred to movement disorder specialists (e.g., dystonia expert) who gave the diagnosis and offered treatment. Respondents reported relief at having a diagnosis but a general lack of understanding of the prognosis and possible options for long-term management; 46.7% said they felt their neurologist did not spend enough time discussing their diagnosis and addressing their concerns. The majority of patients (11/15, 73.3%) said receiving their diagnosis of CD impacted their mental health and eight patients (53.3%) said they had received/were receiving mental health interventions (medication and/or counselling). Survey respondents reported using a variety of alternative sources of information, including the internet (86.7%), self-help groups (66.7%) and information leaflets provided by HCPs (60.0%).

Respondents reported that their neurologists often discussed chemodenervation with BoNT as the main treatment option. While some, but not all, neurologists mentioned complementary treatment approaches such as physiotherapy, they did not consistently refer the patient to the allied services and respondents sought the additional treatments themselves. Survey respondents reported a ‘rollercoaster’ of relief with BoNT treatment with symptoms (and subsequent impact on daily life) returning towards the end of an injection cycle. Respondents noted that *“When BoNT works well I can return to an almost normal life … when the injections stop working so well, I have to rest more and avoid going to work and experience life restrictions.”* A few respondents described experiencing their best effect after their first BoNT injection, and that their CD changed over time *“When the following botulinum toxin injections [after the 1st injection] did not work as well, I lost all hope because I was told it was the only treatment option.”* Three of the survey respondents had opted for a surgical intervention (deep brain stimulation or selective peripheral denervation) because of inadequate relief with BoNT. Finally, survey respondents generally reported acceptance about living with their CD, with some fears for the future – especially as they continue to age. Many respondents described strategies such as looking for social and emotional support (from family, friends, patient groups and professionals), physical exercise, and relaxation strategies as helpful in their day to day lives.

### Expert patient focus group validation of the CDPJM

The expert patient focus group, comprised of patient society representatives living with CD, generally agreed with the findings of the patient survey and the design of the CDJPM. Select quotes from the focus group can be found in Additional file 2. Briefly, the focus group agreed that it can take 2–3 years before a patient receives their diagnosis of CD from a movement disorder specialist. This was discussed as a result of lack of awareness of CD and other rare diseases in primary care. There was general agreement on the importance of patients with CD being referred to a movement disorders expert neurologist for optimal CD management. However, discussion focused on gaps in the communication between the HCP and patient, particularly about the full range of treatment options and what a diagnosis of CD might mean for the patient.

Another potential gap was the lack of a central coordinator between neurology and other (e.g., physiotherapy and psychosocial) support services. In terms of long-term management with BoNT injections, the expert patient focus group agreed with the description of treatment as rollercoaster, where the patient experiences relief following (re)injection and then symptom re-emergence once the effects start to wear-off. The expert patient focus group noted that many clinicians operate injection clinics which are typically too busy to allow for HCP/patient reflection and re-evaluation of treatment. Here, the significant time restraints of a busy injection clinic hinder active participation of the patient who, for example, can worry that if they are perceived as ‘complaining’ that their symptom relief doesn’t last the full injection interval, the injections will be taken away. The busy injection clinic was generally discussed in terms of perpetuating the paternalistic model of medicine – and hindering patient centered care. The worldwide lack of neurologists [24] and the need for better trained injectors [25] were identified as key problems that limit patient access to the right doctors at the right time.

## Discussion

Patient experience and satisfaction have been demonstrated to be the single most important aspect in assessing the quality of healthcare [17]. Accumulating evidence shows the importance of patient engagement and attention to patient expectations in the healing process and it is increasingly accepted that patient involvement in the design of healthcare services improves the relevance and quality of the services [26–28]. This is especially important in the design of services for rare diseases, such as CD, where the knowledge base is often restricted to small numbers of expert doctors. To our knowledge, we present here the first patient journey map for patients living with CD. Importantly, the map was primarily informed by patient experience (in the form of a survey and expert patient focus group) supplemented with clinical guidance and the existing literature.

The mapping process identified five key stages of the patient journey, each with specific gaps in service provision and barriers to optimal care. In stage 1 (symptom onset) family doctor education and awareness were considered the biggest hurdle to diagnosis and, as reported for other rare diseases [29], survey respondents had already seen up to 10 different (non-specialist and specialist) HCPs before being diagnosed with CD by a neurologist. Given the number of rare diseases a family doctor may come across in their daily work, potential solutions to this are difficult but an easier target audience for specific education might be the HCPs to whom the patients are often misdirected (e.g., osteopaths, orthopedics, spine surgeons, physiotherapists etc.). A key gap identified through stages 2 and 3 was the need for improved communication between patient and physician. Here, the mapping process clearly highlighted the need for HCPs to provide their patients with more detailed information on the disease and on the full array of treatment options, including complementary therapies such as physiotherapy and psychosocial support. In such situations, the CDPJM can be used as a tool to help explain a typical clinical pathway to patients and help patients identify their specific needs and raise any issues with their treating team.

With respect to treatment (stages 3–5), survey respondents initially responded they had ‘great hope’ at the start of treatment. This resonates with the results of a prior patient survey which also identified high patient expectations of BoNT treatment, with a majority expecting freedom from spasms and pain and over half expecting to return to a normal routine [14]. Both respondents and the focus group reported a rollercoaster of relief with BoNT treatment with symptoms (and subsequent impact on daily life) returning towards the end of an injection cycle. This strongly aligns with a recent patient survey where Comella and colleagues found that 88% of patients living with CD experience symptom re-emergence that impacts their daily life before the next scheduled injection [15]. Such findings highlight the importance of empowering patients to explain how treatment affects their daily life such that the clinician can work to optimize injection and other treatment parameters for best effect. For example, the early re-emergence of motor symptoms may prompt a reassessment of injection parameters (muscles injected, doses used), while the development of non-motor symptoms such as depression or anxiety may prompt referral to an allied professional. Patients also described that they experienced their best response to BoNT during the first injection cycle(s). This phenomenon is well described, and recent observational studies have shown the greatest symptom relief in newly treated patients [30–32]. However, this does not mean that the treatment is ineffective in chronic patients, and the same studies showed a clinically significant effect and high patient satisfaction (> 80%) across repeat cycles [30]. It has been suggested that the phenomenon we observed in our survey may reflect patient perceptions of their disease and how they self-rate their condition [32]. Our own survey results showed that patients gradually become accustomed to their condition, and it might be that by the time patients are into > 5 years of treatment it may be very difficult for them to remember what it was like before.

The CDPJM identifies several common gaps in service provision. The lack of clear clinical pathways for referrals to physiotherapists and psychologists was identified as a key gap in all participating countries. Here the patient journey map can be used as a baseline tool to understand which HCPs patients find useful, so that movement disorder centers can develop and reinforce links with the allied services such that the long-term management plan for CD becomes much more multidisciplinary. Lessons can be learned from Parkinson’s disease, which is another neurological condition but is far more common than CD [33]. In the UK, Parkinson’s services have traditionally followed a common model of diagnosis by a movement disorder specialist with routine follow-up with a Parkinson’s disease Specialist Nurse who refers back to the specialist as required. However, the importance of a multidisciplinary team approach has been increasingly recognized in this area, and has led to the development of local ‘hub’ services in which a care coordinator serves as the central point of contact coordinating care between established services, including the neurologist, the specialist nurse, physiotherapists, occupational and speech and language therapists etc. [34]. Such integrated care pathways took years to develop but all started with mapping processes based on patient involvement and feedback, similar to the mapping process we present for CD [33, 35].

Although the idea of patient journey maps are gaining traction [17–19], there are no standard approaches to performing the steps of the mapping exercise and it has been suggested that the lack of consistent methodology may contribute to the low adoption rates in healthcare [36, 37]. It is important that our mapping process was patient driven. Although clinicians were involved in the survey design and interpretation of the findings, the CDPJM was purposely designed to reflect the patient perspective; future mapping could look to integrate the healthcare provider point of view. We chose a method which gives the typical patient a ‘persona’ that clinicians and patients can relate to rather than a data set. Although personas are a commonly used tool to help service designers make decisions, they will not capture every individual patient’s needs [38, 39]. In this pilot mapping exercise, we chose to work with smaller groups of patients for ease of communication and because we wanted to collect and collate qualitative feedback. This follows the current recommendations for obtaining deep experiential insights from patients during the mapping process [19, 40]. While larger patient surveys are preferable for collecting quantifiable data, they often miss the unique, connective links that direct patient feedback based on their lived experiences can give. Following this pilot, future work could consider international expansion for a more global approach, or perhaps more pragmatically, a similar mapping process at the national level could also be very informative.

Another possible limitation of the select group of patients involved is the chronicity of their disease. Over half of the patients who responded to the patient survey had been living with their condition for over 10 years, which might have made it more difficult to remember how they felt in the earlier stages of the disease. It is also conceivable that certain processes have changed in the years since their diagnosis. However, we did not observe any obvious quantitative or qualitative differences in the patient experience dependent on the time since diagnosis and the experience of expert patient representatives suggests that little has changed in past decades. Survey respondents and focus group members were all recruited via the participating Dystonia Europe affiliates, which may have introduced bias since people who engage in support groups are often female, younger, more highly educated, of a higher economic status and more anxious about their disease than those who do not [41]. CD is at least twice as common in women than in men [42] and a recent retrospective study at one center found sex differences in the age of onset and treatment response (men were diagnosed younger, had a less robust response to treatment, and were more likely to discontinue care) [43]. As such the male perspective on the patient journey may differ from females and future mapping may benefit from specific subgroup analyses with a larger sample. Another limitation is the small number of participating countries (Italy, France, UK) all of which have public healthcare provision. As such the tool should be considered a baseline that can be tailored to a local hospital, area or region.

In summary, we present the first patient journey map for people living with CD. It is hoped that clinicians interested in the management of CD can use the map as a tool to guide their own service mapping process and compare their services with what patients say they want and need. Similarly, patient societies (including Dystonia Europe and their affiliates) can use the tool to identify gaps in patient education and support networks and identify potential programs in their local areas as potential solutions to unmet needs. A plain language summary of the paper is provided in Additional file 4. These observations have to be carried forward to the relevant organizations devoted to improving patient care (e.g., NIHR in the UK and similar organizations in other countries). As services and treatments evolve, the CDPJM should be re-evaluated and refined over time.

## Methods

### Patient survey

An online survey of 15 patients living with CD was conducted using LamaPoll (see Additional file 3 for patient survey questions). The study was conducted in compliance with relevant codes of conduct from the European Pharmaceutical Market Research Association and the Insights Association (formerly known as CASRO).

The structure and contents of the survey were based on a generic patient mapping survey tailored to CD in collaboration with patient representatives from Dystonia Europe, representatives from the European Reference Network for Rare Neurological Diseases, sponsor representatives and experts from the patient experience company. All 15 patients were recruited by the participating Dystonia Europe affiliates (France: AMADYS; Italy: Associazione per la Ricerca sulla Distonia A.R.D.; United Kingdom: Diagnosis, Education and Research [ADDER]). Other than having a diagnosis of CD (self-reported), there were no formal inclusion or exclusion criteria for participation in the mapping process. The survey was conducted in English and included 45 questions. Questions were a mix of multiple choice and free entry formats, and data analysis was purely descriptive.

### Developing and validating the CDPJM

To support the development of the CDPJM, a broad literature review was performed using PubMed and Google Scholar to identify relevant literature, search terms included cervical dystonia OR spasmodic torticollis AND diagnosis, treatment, patient. References were limited to those published between 2000 and 2021 and those published in English or German. Using the survey results, a first draft patient journey map was developed, and sense checked against the current literature. This first draft map was then shared with expert patients from Dystonia Europe and its participating affiliates, and an online focus group meeting was convened in May 2021 to come to consensus on each of the stages identified in the mapping process. Focus group participants reviewed each stage of the CDPJM and had an open discussion on how well the map reflects the care pathway in the countries they represent. The focus group meeting was recorded, and the meeting minutes were used to refine the map into the final CDPJM.

## Supplementary Information

**Additional file 1.**. Full version of the CD patient journey map.

**Additional file 2.** Verbatim quotes from the expert focus group.

**Additional file 3.** Patient survey.

**Additional file 4.** Plain language summary.

## Data Availability

The CD patient journey map is available on request from the corresponding author.

## Abbreviations

BoNT: Botulinum neurotoxin
CD: Cervical dystonia
CDPJM: Cervical dystonia patient journey map
