# Supplementary figures and images for: Development of a patient journey map for people living with cervical dystonia
Source: Orphanet J Rare Dis. 2022 Mar 21;17:130. doi: 10.1186/s13023-022-02270-4 (PMC8935780; doi:10.1186/s13023-022-02270-4)

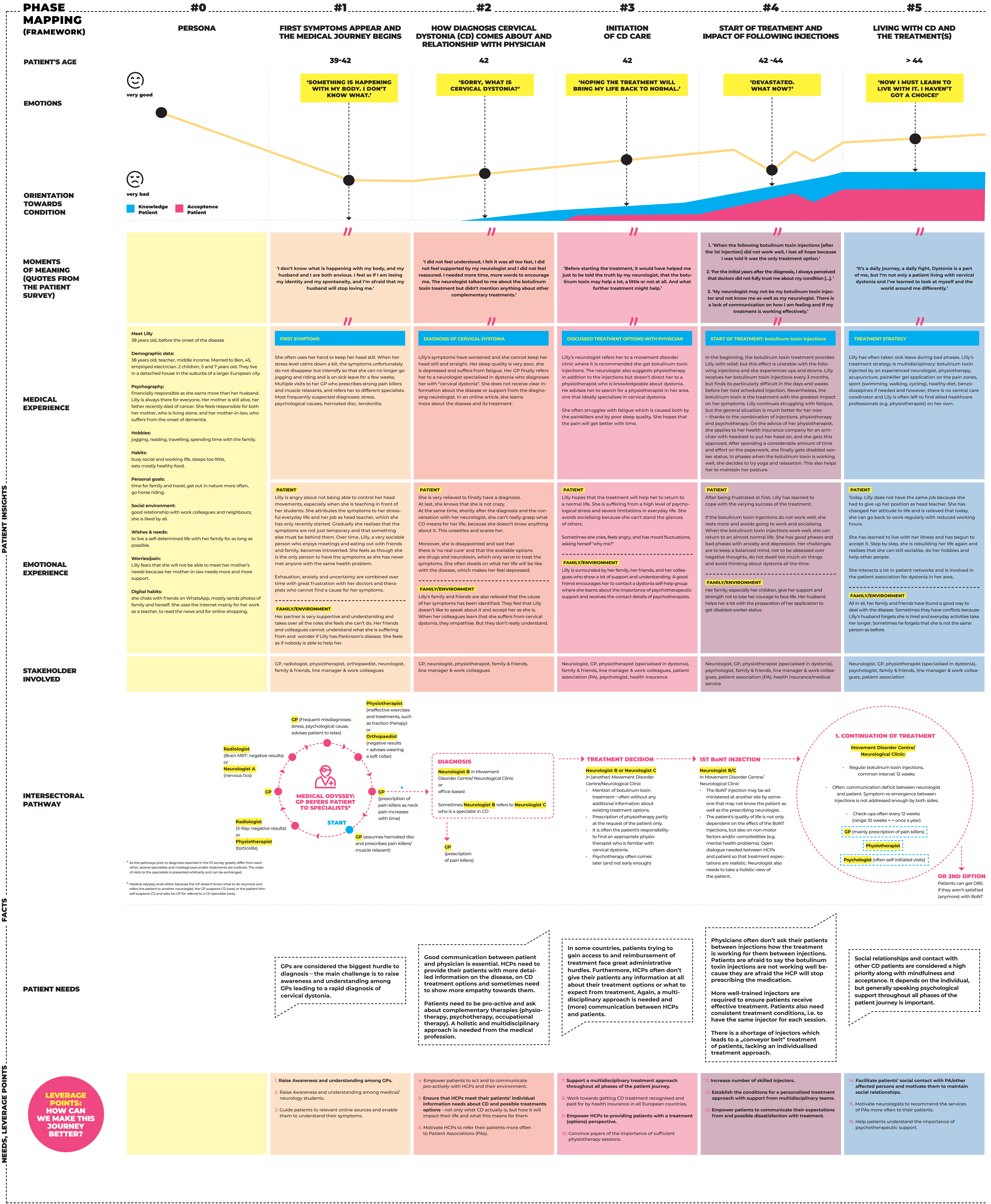

Supplement: Supplementary file 1 — Additional file 1.. Full version of the CD patient journey map. [file 13023_2022_2270_MOESM1_ESM.pdf]
