# Supplementary material for: Development of a patient journey map for people living with cervical dystonia
Source: Orphanet J Rare Dis. 2022 Mar 21;17:130. doi: 10.1186/s13023-022-02270-4 (PMC8935780; doi:10.1186/s13023-022-02270-4)
Supplement: Supplementary file 2 — Additional file 2. Verbatim quotes from the expert focus group. [file 13023_2022_2270_MOESM2_ESM.pdf]

Supplementary Appendix 2.

Expert Patient Focus Group Verbatim Quotes

**Symptom onset and diagnosis**
In France, General Practitioners (GPs) are not well informed of all the rare diseases.
I went to the GP, and she didn't know what it was and then I went home and I Googled things like 'head twisting' and 'muscles pulling' to see if I could get the information on the internet.
It's important for patients to be able to contact patient organizations and where to find good, reliable information online because there is a lot of misinformation around.
Once you get the neurologist of the center which is concerned with movement disorders you are finally in the right place [diagnosis and treatment]
In some parts of the UK, the neurologists are all very knowledgeable about dystonia and there are other areas of the UK where they are not so knowledgeable. What tends to happen in those areas is that the patient does some research to find who they should be referred to for the best treatment.
As soon as you are diagnosed in the UK you have access to treatment, but there is no such thing as a rapid diagnosis.

**Initiation of treatment and the therapeutic relationship with HCPs**
It is important that the physician gives adequate information at the first time of treatment and diagnosis, maybe not all the details, but the patients have a right and they need to know, because I didn't find out anything when I had my first botulinum toxin injection. My doctor only said 'I hope this will work for you'. I went home and I looked at the internet and there I saw that dystonia was a chronic disease and I started to cry in front of the computer.
Usually, [the neurologists] don't give enough details [at diagnosis]. They just give general information, but they don't spend time in explaining 'Now you are going to be injected, but success depends on injecting the correct muscles and dosing etc.'.
Maybe at the beginning the neurologists feel they don't want to overwhelm the patient with information, so maybe this is a classic miscommunication because the doctor thinks *'I don't want to throw all of this information about what might happen in the future… We need to tell them that we need more information'.*
There is a need for patients to have a helicopter view of how CD is treated generally and what are all the options that are available.
in the UK, we see a specialist for ten minutes once every three months, and in the early days that's just not enough.
In the UK, patients don't have immediate access to something like physiotherapy or any other complementary therapy. The patient has to look for it themselves.

In Italy, patients must look for physiotherapies themself because the neurologist doesn't give the information. You just feel like a neck to be injected and that's all except they give the next appointment. Some specialist centers do have the connections between the neurologist, the physiotherapist and the psychologist, but they are in just one city with an expert CD doctor.
I am afraid specialists don't know each other between specialties. Most of the time neurologists don't know the physiotherapists and which one is able to take care of dystonia. Neurologists often look to patient organization listings to know the physiotherapists around them able to take care of their patients, so what is the matter? They need to be connected.
In France, there are not many physiotherapists who know about dystonia.
The only time I see my GP in relation to my dystonia is if my consultant has recommended some drug therapy and he would write to my GP. I would then make an appointment to see my GP and they would prescribe the drugs.

**Living with treated CD (impact of BoNT-A injections)**
Personally, I couldn't move on with my life until I had accepted dystonia into my life, and it was a very important moment for me when that happened.
Injection clinics are planned to inject, 10-15 people per session, so they don't have time to talk. If you want to talk about your disease effects, the evolution, or whatever, you need to take an appointment with your neurologist outside these days and have time to discuss, but this is not well understood by most patients.
Many patients are not fully satisfied with their injections, but they are afraid to tell their consultant in case the consultant says, "I will just stop giving them to you, then." A lot of patients have that fear.
Another problem is there are not enough neurologists around, which limits patient access to the right doctors.
