# Supplementary material for: Development of a patient journey map for people living with cervical dystonia
Source: Orphanet J Rare Dis. 2022 Mar 21;17:130. doi: 10.1186/s13023-022-02270-4 (PMC8935780; doi:10.1186/s13023-022-02270-4)
Supplement: Supplementary file 3 — Additional file 3. Patient survey. [file 13023_2022_2270_MOESM3_ESM.pdf]

Supplementary Appendix 3

Patient Survey

PERSONAL DETAILS

1. Initials:
2. Sex:
3. Age:
4. Country you live in: France/Italy/UK
5. Are you employed?

    If answer = Yes: full-time, self-employed, part-time, full-time student

    If answer = No : retired, not able to work/disabled, currently not employed
6. Did you have to change your job because of Cervical Dystonia?

    Option box: yes / no / other
7. What is the distance from your place of residence to medical centers (specialists, clinics, university centers)?

    Option box: < 5 km, 5-20 km, 20 -50 km, 50 – 100 km, > 100 km

BEFORE DIAGNOSIS

The following questions refer to your life before diagnosis. We therefore ask you to remember what your life was like before diagnosis, but when you already had symptoms of cervical dystonia.

8. Could you please confirm when you first noticed symptoms that could subsequently be attributed to Cervical Dystonia?

    Month: Year:
9. What symptoms did you have exactly?
10. How often did you experience these symptoms?

    Option box: very frequently, frequently, occasionally, rarely
11. Were any of your bodily functions impaired at the time (e.g. eating, drinking, walking, any physical activities, etc.)

    Option box: yes / no

    If yes, which ones?

    Do these impairments still exist today?

    Option box: yes / no
12. Please describe how the symptoms impacted on your life?

    Family: Partnership: Job: Interests and hobbies: Mental health:
13. Did the symptoms affect your quality of sleep?

    Option box: yes / no

    If yes, do you still suffer from sleep problems today?

    Option box: yes / no

14. How did you feel during this time when you had symptoms but no diagnosis?

DIAGNOSIS

15. When were you diagnosed with Cervical Dystonia?

    Month: Year: Your age at this time:

16. How did the diagnosis affect you? What went through your mind?
17. When your symptoms first started, what healthcare providers did you consult before finally being diagnosed (e.g., GP, neurologist, physiotherapist)?

    Specialty of the healthcare provider → Outcome of the consultation (in terms of diagnosis and treatment)

18. Can you remember after approximately how many visits* at which healthcare provider you were diagnosed with Cervical Dystonia?

    *Please count several visits to the same physician/ therapist e.g. 2 visits to a neurologist and 1 visit to a physiotherapist) = 3 visits.

    Specialty of healthcare provider(s) → number of visits:

19. Were there any misdiagnoses? If yes, which ones?
20. Did you also receive incorrect treatments due to these misdiagnoses? if yes, which ones?
21. What physician/healthcare provider actually made the final diagnosis?

    Option box: neurologist, physiotherapist, other: [free text field])

22. Did you know about Cervical Dystonia before being diagnosed?

    Option box: yes / no /

23. Do you feel your healthcare professional spent enough time discussing your diagnosis and addressing your concerns?

    Option box: yes / no / other

24. Were you satisfied with the information you received from your healthcare professional?

    Option box: yes / no /

    If not, please explain why you were dissatisfied.

25. How did you feel after the appointment? (For example, quite hopeful, lost, left alone, well supported?)
26. What would have helped to make you feel better?
27. Did the diagnosis of cervical Dystonia have an impact on your mental health?

    Option box: yes / no / I prefer not to answer

    If yes: When did you notice a change in your mental health?

    a) Are you still suffering from it today?

    Option box: yes / no / I prefer not to answer

    b) Have you been or are you receiving medical care for this?

    Option box: yes / no

    If yes: medication: yes/no

    Counselling: yes/no

28. Did you suffer from other conditions triggered by Cervical Dystonia?

Option box: yes / no / I prefer not to answer

If yes, which ones?

a) At what stage of your diagnosis did this occur?

Option box: within 1 year, within 1-5 years, > 5 years

b) Do these still exist today?

Option box: yes / no

c) Have you been or are you receiving medical care for this?

Option box: yes / no

TREATMENT

29. Who is/was your attending healthcare provider after the diagnosis? (e.g. general practitioner, neurologist)

30. Which treatment options were discussed with you?

31. What treatment did you receive/are you receiving? (Please list chronologically all therapies received)

    If several treatment options = yes: Did your doctor involve you in the decision for or against a certain treatment?

    Option box: Yes/ no/ other

32. Are you currently receiving a treatment?

    Option box: yes / no

    If yes: which one?

33. Does or did the treatment have an impact on your life/your everyday life? (Family, partnership, job, interests and hobbies...)?

    Option box: yes / no

    If yes: please describe what impact the therapy has or had.

34. Please think back to the time when you were diagnosed and possibly received a certain medication and/or treatment:

    a) How did you feel at that time?

    b) Was there anything that particularly helped you to cope better with Cervical Dystonia?

YOUR LIFE WITH CERVICAL DYSTONIA

35. How often do you go for a check-up?

    a) What is done during these check-ups?

36. What healthcare providers do you see for treatment of the symptoms today?

37. Have your symptoms changed over time?

    Option box: yes / no

    If yes: Please describe how they have changed?

38. What challenges does Cervical Dystonia pose in everyday life?

    Family: Partnership: Job: Interests and hobbies: Mental health:

39. When it was clear that you had Cervical Dystonia: Did you look for further information about it?

Option box: yes / no

If yes, where?

On the internet/ Self-help groups / Patient Organizations/ in patient brochures provided to me by: my physician / my attending clinic / during a stay in rehabilitation / a website

Other, namely:

40. Looking back, what do you wish had been different regarding your experience with Cervical Dystonia?

a) What would have helped you? (e.g. special contacts, special services)

41. Do you have any specific coping strategies to manage your Cervical Dystonia symptoms?

Option box: yes / no

If yes, which ones?

42. Is there anything specific that improves your Cervical Dystonia symptoms?

Option box: yes / no

If yes, please explain

43. Is there anything specific that worsens your Cervical Dystonia symptoms?

Option box: yes / no

If yes, please explain

44. How do you feel about your disease today?

45. What would you like to achieve for yourself? What timeframe?
