# Supplementary material for: Development of a patient journey map for people living with cervical dystonia
Source: Orphanet J Rare Dis. 2022 Mar 21;17:130. doi: 10.1186/s13023-022-02270-4 (PMC8935780; doi:10.1186/s13023-022-02270-4)
Supplement: Supplementary file 4 — Additional file 4. Plain language summary. [file 13023_2022_2270_MOESM4_ESM.pdf]

**Appendix 4**

**Lay summary**

**Development of a patient journey map for people living with cervical dystonia**

Monika Benson,[1,2] Alberto Albanese,[2,3] Kailash Bhatia,[4] Pascale Cavillon,[5] Lorraine Cuffe,[6]
Kathrin König,[7] Carola Reinhard,[2,8] Holm Graessner[2,8]

**Plain language summary**

It is known that treatment always work best when the patients actively participate in decisions relating to their own healthcare. Patient journey maps are one tool that healthcare services can use to visualize the long-term care of their patients. To help the professionals understand all the small steps a person with a rare disease goes through, from their first symptoms through to diagnosis and eventual treatment. A collaborative group of experts, including consultants and people living with cervical dystonia (often shortened to CD), set out to develop a map that visually explained the whole disease journey ***from the patients point of view***.

The group found that people living with CD typically go through five key stages. Using detailed feedback from patients they developed a map which described the typical patient journey.

1. Symptom onset
2. Diagnosis and therapeutic relationship with healthcare professional (HCPs)
3. Initiation of care for CD
4. Start of CD treatment
5. Living with treated CD

The process of building this visual map identified several barriers to good treatment in CD. For example, misdiagnoses, lack of care coordination and ineffective communication between patients and their doctors. By developing the first patient journey map for CD, the authors hope that doctors can use the map as a tool to improve the care they offer. Patients can also use the tool as a visual aid to help them describe their experience, including any concerns with long-term treatment plans, to the healthcare professionals using a common language.
